# Supplementary material for: Integrated Metabolomic and Transcriptomic Analyses of the Flavonoid Biosynthetic Pathway in Relation to Color Mutation in Roses
Source: Biology (Basel). 2025 Sep 29;14(10):1337. doi: 10.3390/biology14101337 (PMC12561831; doi:10.3390/biology14101337)
Supplement: Supplementary file 1 [file biology-14-01337-s001.zip › biology-3860397-supplementary.pdf]

Supplementary information

Table S1. Functional classification of metabolites identified in rose petals.

Type	Number	Percentage
all	479	100
Flavonols	183	38.20
Flavones	104	21.71
Flavanones	24	5.01
Tannins	47	9.81
Flavanols	40	8.35
Aurones	12	2.51
Chalcones	15	3.13
Isoflavones	10	2.09
Flavanonols	4	0.84
Anthocyanins	12	2.51
Proanthocyanins	18	3.76
Other flavonoids	10	2.09

Table S2. 255 differentially accumulated metabolites in the petals of AR and SR.

Class	Metabolite	Content		Log2(FC)
		AR_Mean	SR_Mean	
Quercetin and derivatives	Quercetin	5.74E+04	1.73E+05	-1.59
	Dihydroquercetin (Taxifolin)	1.89E+04	2.74E+05	-3.86
	Quercetin-3,4'-O-di-glucoside	3.61E+04	1.85E+05	-2.36
	Quercetin 3-O-caffeyl-glucoside	4.63E+04	3.20E+05	-2.79
	Quercetin-3-O-(2"-O-galloyl) galactoside	1.60E+06	7.73E+06	-2.27
	Quercetin-3-O-(6"-O-galloyl) glucoside	2.05E+06	8.86E+06	-2.11
	Quercetin-3-O-(2"-O-galactosyl) glucoside	1.68E+04	1.21E+05	-2.85
	Quercetin-3-O-(6"-O-p-Coumaroyl) glucoside	1.25E+06	2.88E+06	-1.21
	Quercetin-3-O-(6"-O-p-Coumaroyl) galactoside	1.35E+06	3.01E+06	-1.16
	Quercetin-3'-(6"-O-p-Coumaroyl) glucoside	1.30E+06	2.70E+06	-1.06
	Quercetin-3-O-(2"-O-p-Coumaroyl) galactoside	1.70E+05	4.05E+05	-1.25
	Quercetin-3-O-(2"-O-p-Coumaroyl) glucoside	1.60E+05	3.68E+05	-1.20
	Quercetin 7-O-p-coumaroyl rhamnoside	1.63E+04	4.92E+04	-1.59
	Quercetin-3-O-(4"-O-galloyl) arabinoside	6.84E+04	1.27E+06	-4.22
	Quercetin-3-O-α-rhamnosyl (1→2)-[α-rhamnosyl (1→6)]-β-glucoside	1.14E+04	1.34E+05	-3.56
	Quercetin-3-O-(2"-O-galloyl) Arabinoside	8.61E+04	1.27E+06	-3.88
	Quercetin-3-O-(2"-O-cinnamoyl) Glucoside	3.13E+05	1.27E+06	-2.03
	Isoquercitrin	3.10E+07	5.96E+06	2.38
	Quercetin-3,7-Di-O-rhamnoside	7.92E+06	6.91E+05	3.52
	Quercetin-3-O-neohesperidoside	1.08E+06	5.33E+05	1.02
	2"-O-Galloylquercitrin	3.59E+07	4.77E+06	2.91
	3"-(E)-Cinnamoylquercitrin	1.70E+06	3.30E+05	2.36
	7,3',4'-Trihydroxyquercetin	1.06E+05	1.36E+04	2.95
	Quercetin 3-(6"-feruloylgalactoside)	9.15E+04	4.41E+04	1.05
	3-O-Methylquercetin-3-O-(6"-malonyl) glucoside	5.22E+05	3.46E+04	3.91
	Quercetin-3-O-(6"-O-acetyl) galactoside	1.32E+06	4.75E+05	1.48
	Quercetin 3-O-(6-O-malonyl-beta-D-glucoside)	3.61E+05	1.17E+05	1.63
	Quercetin-3-O-(6"-O-malonyl) glucosyl-5-O-glucoside	2.84E+05	8.59E+03	5.05
	Quercetin 3-O-(6'-malonyl-glucoside) 7-O-glucoside	8.29E+05	2.82E+04	4.88
	Multinoside A	2.66E+05	2.15E+04	3.63
	Quercetagetin-7-O-glucoside	1.03E+06	4.09E+05	1.33
Kaempferol and derivatives	kaempferol 5-O-glucuronide	1.51E+05	2.17E+06	-3.85
	6-Hydroxykaempferol 3-glucoside	8.42E+06	1.99E+07	-1.24
	8-C-Methylkaempferol 7-glucoside	1.57E+05	7.02E+06	-5.48
	Kaempferol 5-methyl ether 3-galactoside-4'-glucoside	5.45E+04	4.40E+05	-3.01
	Kaempferol-3-(6"-benzoylgentiobioside)	4.71E+04	3.26E+05	-2.79
	Kaempferol 3-(6"-caffeoylglucoside)	1.60E+06	1.64E+07	-3.35
	Kaempferol-3-O-[(6'''-p-coumaroyl)-3-O-D-glucosyl]-glucoside	3.38E+03	3.18E+04	-3.23
	Kaempferol 3-[6"-(3-hydroxy-3-methylglutaryl)glucoside	9.86E+04	6.92E+05	-2.81
	Kaempferol-3-O-(6"-Rhamnosyl-2"-Glucosyl)Glucoside	2.58E+04	2.26E+05	-3.13
	Kaempferol-3-O-glucorhamnoside	2.78E+07	5.83E+07	-1.07

Continued

	**Kaempferol-7-O-(6''-O-p-caffeoyl)glucoside**	**1.85E+06**	**2.08E+07**	**-3.50**
Kaempferol and derivates	Kaempferol 3-sophorotrioside	1.78E+04	4.52E+04	-1.35
	kaempferol-3-O-β-glucopyranosyl (1 → 2)-β-galactopyranoside 7-O-α-rhamnopyranoside	1.14E+04	1.12E+05	-3.29
	Kaempferol	9.06E+05	4.68E+04	4.28
	6,8-Di-C-methylkaempferol 7-methyl ether	6.76E+04	2.96E+03	4.51
	Kaempferol-3-O-(6''-p-Coumaroyl)galactoside	4.56E+06	1.99E+05	4.52
	Kaempferol 3-(6''-(Z)-cinnamylglucoside)	1.80E+06	1.51E+05	3.57
	Castanoside A[Kaempferol-3-O-(6''-p-coumaroyl)mannoside]	1.05E+06	1.21E+05	3.12
	Kaempferol-3-O-Apiosyl-(1→2)-Galactoside	1.31E+07	1.38E+06	3.25
	Kaempferol 3-xylosylglucoside	1.51E+07	1.58E+06	3.26
	Dihydrokaempferol-3-O-glucoside	1.72E+07	2.26E+06	2.93
	Kaempferol-3-O-(6''-malonyl)glucoside	2.41E+07	2.44E+06	3.30
	Kaempferol-3-O-(6''-malonyl)galactoside	2.23E+07	2.34E+06	3.25
	Kaempferol-3-O-(2''-galloyl)glucoside	2.98E+07	3.09E+06	3.27
	Kaempferol 7-glucosyl-(1->4)-xyloside	2.09E+07	4.47E+06	2.22
	Kaempferol-3-O-(6''-galloyl)glucoside	6.31E+07	2.46E+07	1.36
	Kaempferol 7-(6''-galloylglucoside)	7.25E+07	2.85E+07	1.35
	Kaempferol-3-O-(6''-galloyl)galactoside	6.24E+07	2.47E+07	1.33
	Kaempferol-3-O-(2''-galloyl)galactoside	7.76E+07	2.37E+07	1.71
	Kaempferol-3-(2'',6''-di-O-rhamnosyl)-glucoside	3.45E+05	1.48E+04	4.54
	Kaempferol 3-rhamnosyl-(1->2)-glucosyl-(1->6)-galactoside	1.32E+05	2.05E+04	2.69
	Kaempferol-3-O-(6''-Malonyl)glucoside-7-O-Glucoside	1.54E+06	3.06E+04	5.66
	kaempferol 3-O-(6'-O-feruloyl)-glucoside	3.28E+05	1.64E+04	4.32
	Kaempferol 3-apiosyl-(1->2)-alpha-L-arabinofuranoside-7-rhamnoside	6.96E+05	1.78E+04	5.29
	Kaempferol-3-O-rutinoside-7-O-rhamnoside	6.53E+05	1.38E+04	5.57
	8-methoxykaempferol 3-O-(6''-malonyl-β-glucopyranoside)	6.43E+05	4.07E+04	3.98
	kaempferol 3-O-(2''-O-malonyl)glucoside	1.95E+06	6.50E+05	1.58
	Kaempferol-3-O-(2''-p-Coumaroyl)galactoside	1.46E+06	1.62E+05	3.16
	Kaempferol-3-(p-benzoyl)glucoside	7.80E+04	8.47E+03	3.20
	Kaempferol 3-(2G-rhamnosylgentiobioside)	1.25E+05	2.48E+04	2.34
	Kaempferol-3-O-sophoroside-7-O-rhamnoside	1.36E+05	1.46E+04	3.22
	Kaempferol-3-O-neohesperidoside-7-O-glucoside	1.35E+05	1.88E+04	2.85
	Kaempferol-3-O-rhamninoside	6.06E+05	3.62E+04	4.06
	6-Hydroxykaempferol 3-methyl ether 6-glucoside	6.32E+04	4.97E+03	3.67
	Kaempferol-3-O-(2''-p-Coumaroyl)glucoside	4.81E+05	5.25E+04	3.20
	Kaempferol-3-O-(2''-O-acetyl)glucoside	1.84E+07	9.30E+05	4.30
	Kaempferol-7-O-glucoside	4.68E+07	1.37E+07	1.77
	Kaempferol-3-O-galactoside	3.36E+07	5.77E+06	2.54
	Kaempferol-3-O-(2'',6''-digalloyl)glucoside	6.64E+06	1.12E+06	2.56
	Kaempferol-3-O-robinobioside (Biorobin)	3.30E+07	2.85E+06	3.53
	6-Methoxykaempferol-3-O-glucoside	2.20E+05	8.75E+04	1.33
	Kaempferol 3-(4''-p-coumaroylglucoside)	9.73E+06	1.02E+06	3.25

Continued

	**Kaempferol-3-O-arabinoside**	**7.53E+07**	**1.91E+07**	**1.98**
	Kaempferol-3-O-rhamnosyl(1→2) glucoside	1.01E+07	8.86E+05	3.51
	Kaempferol-3-O-(6''-O-acetyl) glucoside	9.55E+06	6.52E+05	3.87
	Kaempferol-3-O-glucoside-7-O-rhamnoside	3.08E+07	6.47E+04	8.89
	Kaempferol-3-O-(4''-O-p-Coumaroyl) rhamnoside	4.24E+04	6.44E+03	2.72
	Kaempferol-3,7-O-diglucoside	2.05E+05	2.13E+04	3.27
	Kaempferol-3-O-rutinoside	1.12E+07	6.45E+05	4.11
	Kaempferol-3-O-neohesperidoside	1.44E+06	9.62E+04	3.91
**Luteolin and derivates**	Luteolin	8.87E+05	4.32E+04	4.36
	Luteolin-7-O-(6''-malonyl)glucoside-5-O-rhamnoside	2.79E+05	1.80E+04	3.95
	Luteolin 6-C-Arabinoside-4'-O-Rhamnoside	5.79E+05	4.37E+04	3.73
	Luteolin 7-O-(6''-malonylglucoside)	2.83E+07	2.96E+06	3.26
	Luteolin 7-rutinoside-4'-glucoside	5.69E+05	5.13E+04	3.47
	Luteolin 7-(6''-p-benzoylglucoside)	2.95E+05	1.57E+04	4.24
	Luteolin 7-(6''-ferulylglucoside)	1.14E+05	7.90E+03	3.85
	Luteolin 7-(6''-acetylglucoside)	1.89E+07	1.15E+06	4.04
	Luteolin 7-O-p-coumaroyl rhamnoside	4.29E+04	9.23E+03	2.22
	Luteolin 5-O-p-coumaroyl rhamnoside	5.11E+04	7.08E+03	2.85
	8-Methoxyluteolin-8-glucoside	7.56E+05	3.38E+05	1.16
	6-Hydroxyluteolin 6-xyloside	4.50E+05	1.46E+06	-1.70
	Luteolin-7-glucuronide ethyl ester	7.43E+04	2.07E+05	-1.48
	6-Hydroxyluteolin 6-rhamnoside	4.85E+07	9.90E+07	-1.03
	Luteolin 3'-methyl ether 5,4'-diglucoside	3.47E+04	3.65E+06	-6.71
	6-Hydroxyluteolin-6-O-(2''-O-p-Coumaroyl) galactoside	1.32E+05	3.36E+05	-1.35
	Naringenin-4'-O-glucoside	1.81E+05	7.50E+05	-2.05
**Catechin derivates**	(+)-Gallocatechin	4.93E+06	2.17E+06	1.19
	Robinetinidol-(4alpha->8)-catechin-(6->4alpha)-robinetinidol	6.11E+05	1.68E+06	-1.46
	Dehydrodicatechin A	1.46E+05	3.60E+05	-1.30
	Catechin gallate	6.87E+05	1.64E+06	-1.25
	2α,3α-Epoxy-5,7,3',4'-tetrahydroxyflavan-(4β→8)-catechin	3.80E+04	3.97E+05	-3.38
	Catechin-(4α→8)-catechin-(4α→6)-catechin	2.10E+04	7.12E+04	-1.76
**Epicatechin derivates**	Epicatechin-(3'-O-7'')-epiafzelechin	5.21E+04	2.07E+04	1.33
	Tetrahydroxyflavan-(4α-8-epicatechin)	3.73E+04	3.95E+05	-3.41
	2α,3α-Epoxy-5,7,3',4'-tetrahydroxyflavan-(4β→8)-epicatechin	2.74E+04	2.45E+05	-3.16
	Epicatechin 3',5-Digallate	9.22E+05	2.05E+06	-1.15
	Epicatechin 4',5-Digallate	8.99E+05	2.13E+06	-1.24
	Epicatechin 3,5-Digallate	8.85E+05	4.74E+06	-2.42
	9,10-Dihydro-10-(4-hydroxyphenyl)-pyrano epicatechin-8-one gallate	1.84E+05	2.70E+06	-3.88
**Apigenin derivates**	8-Hydroxyapigenin 8-(6''-E-p-coumaroylglucoside)	1.65E+07	1.16E+06	3.83
	Vicenin-2	8.27E+04	2.00E+04	2.05
	Apigenin-6-C-(2''-rhamnosyl) glucoside	4.95E+05	7.76E+04	2.67
**Scutellarein derivates**	Scutellarein-6-(2''-coumaroyl) glucoside	1.10E+06	1.33E+05	3.05
	Scutellarein 6-xyloside	9.71E+06	2.47E+06	1.97

Continued

	**Isoscutellarein 8,4'-dimethyl-7-ether glucoside**	**1.10E+05**	**3.26E+04**	**1.76**
**Scutellarein derivates**	Scutellarein 7-(6''-malonylglucoside)	1.86E+06	6.51E+05	1.52
	Scutellarein-7-O-glucuronide (Scutellarin)	1.63E+05	4.35E+04	1.91
	Scutellarein 7-[6''-(3-Hydroxy-3-methylglutaryl) glucoside	1.43E+05	2.14E+06	-3.90
	Scutellarein 7-xylosyl-(1->4) rhamnoside	9.72E+04	1.09E+04	3.16
	baicalein 6-O-β-D-glucuronide	1.94E+05	2.13E+04	3.19
	Scutellarein 5-glucuronide	1.45E+05	2.32E+06	-4.00
**Orientin derivates**	Orientin-2''-O-rhamnoside	4.07E+07	1.76E+07	1.21
	Orientin-7-O-arabinoside	3.27E+05	7.52E+04	2.12
	Orientin 2''-O-beta-L-arabinofuranoside	7.15E+06	1.71E+06	2.07
	Orientin-2''-O-(6'''-p-coumaroyl)-glucopyranoside	1.38E+04	1.16E+05	-3.08
Phlorizin and **derivates**	Phloretin 3',5'-Di-C-glucoside	8.08E+05	2.88E+05	1.49
	Phlorizin chalcone	3.50E+04	5.19E+05	-3.89
	Phlorizin	3.46E+05	1.97E+06	-2.51
**Myricetin derivates**	Myricetin-3-O-β-D-glucoside	8.92E+05	3.81E+05	1.23
	Myricetin-3-O-galactoside	5.78E+05	2.51E+05	1.20
	Myricetin-3-O-rutinoside	5.26E+04	7.22E+03	2.86
	Myricetin-3-O-galactoside-3'-O-rhamnoside	7.18E+04	9.66E+03	2.89
	Myricetin 7-(6''-galloylglucoside)	3.66E+05	3.32E+04	3.47
	Cannabiscitrin	4.69E+04	1.03E+05	-1.13
**Nevadensin derivates**	Nevadensin 7-glucoside	3.65E+05	3.33E+04	3.46
	Nevadensin	4.42E+04	3.32E+03	3.74
**Chrysin derivates**	Chrysin-5-O-glucoside (Toringin)	2.23E+05	5.01E+05	-1.17
**Rhamnetin derivates**	Rhamnetin-3-O-Glucoside	6.46E+05	2.86E+05	1.18
	6-Hydroxyrhamnocitrin 3-O-glucoside	6.64E+05	3.11E+05	1.09
	Tricetin 5-(4''-galloylrhamnoside)	2.00E+06	8.76E+06	-2.13
	Isorhamnetin 3-(2''-acetylglucoside)	1.71E+05	1.18E+04	3.86
**Morin derivates**	Morin-3-O-xyloside	4.81E+07	1.08E+08	-1.17
	Morin	1.04E+06	2.59E+06	-1.31
	Morin 3,7,4'-trimethyl ether 2'-glucoside	3.38E+05	3.17E+04	3.41
	Eriodictyol-7-O-glucoside	1.74E+07	2.20E+06	2.98
	Nevadensin	4.42E+04	3.32E+03	3.74
	Casuarictin	3.58E+05	1.59E+05	1.17
	Neosakuranin	4.44E+07	9.20E+06	2.27
	4'-demethyl-3,9-dihydroeucomin glucoside	4.18E+07	1.00E+07	2.06
	Carthamone	3.78E+07	9.01E+06	2.07
	Plantaginin	4.89E+07	1.03E+07	2.25
	Norartocarpetin	7.33E+06	2.44E+05	4.91
	Okanin4'-(6''-acetylglucoside)	3.42E+05	1.21E+04	4.82
	Pinocembrin 7-rhamnosylglucoside	2.61E+04	2.07E+03	3.65
	Maritimetin	1.82E+05	5.75E+03	4.98
	Gambiriin B2	1.81E+05	1.53E+04	3.57
	Marein	8.65E+05	1.11E+05	2.96

Continued

**Sanguiin H7**	**7.30E+05**	**3.24E+05**	**1.17**
Velutin (5,4'-dihydroxy-7,3'-dimethoxyflavone)	2.29E+04	1.91E+03	3.58
Nepetin-7-O-glucoside(Nepitrin)	2.10E+05	5.55E+04	1.92
Calyxanthone	6.99E+06	3.00E+05	4.54
Gambiriin B1	8.14E+05	2.74E+05	1.57
Dihydroxy-dimethoxyflavone-7-O-glucoside	1.20E+05	3.75E+04	1.68
Pachypodol	1.14E+05	1.63E+04	2.81
Fisetin	2.84E+06	9.69E+05	1.55
5,7,3',4'-Tetrahydroxy-8-methoxyflavone	4.89E+06	9.87E+04	5.63
Gossypetin-3-O-glucoside	9.52E+05	3.03E+05	1.65
Ellagic acid pentoside	7.54E+06	3.29E+06	1.20
Chiirirhamnin	2.61E+05	5.17E+04	2.34
3',5,5',7-Tetrahydroxyflavanone-7-O-glucoside	2.13E+07	3.57E+06	2.58
Buddlenoid A	1.42E+06	1.66E+05	3.10
3'-O-Methyltricetin-5-O-glucoside	4.31E+06	5.43E+04	6.31
Tamarixetin-3-O-(6''-malonyl)glucoside	5.68E+05	2.70E+04	4.40
2'-Hydroxygenistein	2.06E+06	8.37E+05	1.30
3'-O-Methyltricetin	2.35E+06	4.41E+04	5.73
3',4',5,5',6,7-hexahydroxyflavone 6-glucoside	3.39E+04	3.05E+03	3.48
Pedaliin	3.22E+06	2.72E+05	3.56
Nevadensin 7-glucoside	3.65E+05	3.33E+04	3.46
3',5'-dihydroxy-5,7,4'-trimethoxyflavone-3'-glucoside	3.07E+05	3.05E+04	3.33
Tupichinol E	5.32E+04	5.61E+03	3.25
Pedunculagin	2.02E+06	8.51E+05	1.24
6,7,3',4'-Tetrahydroxyaurone-6-O-(6''-O-p-Coumaroyl)glucoside	4.57E+06	3.13E+05	3.87
3-(3,4-Dihydroxyphenyl)-1-(3,4,5-trihydroxyphenyl) prop-2-en-1-one	1.10E+05	1.44E+04	2.94
4',5,6,7-Tetramethoxyflavone	2.18E+04	8.16E+04	-2.72
5,6,7,8-Tetramethoxyflavone	1.90E+04	6.33E+04	-1.64
4,6,4'-Trihydroxyaurone 4,6-di-O-glucoside	8.71E+06	6.53E+07	-6.19
Tangeretin	4.14E+04	8.81E+04	-5.70
3',4',5',5,7-Pentamethoxyflavone	2.77E+04	6.29E+04	-1.90
6-Hydroxygenistein-6,7-diglucoside	2.20E+05	2.64E+07	-3.76
Arecatannin B1	1.09E+05	2.95E+05	-1.82
3,5,6,7-Tetramethoxyflavone	1.89E+04	6.43E+04	-1.30
7,3',4'-Trihydroxyflavone 7-glucoside	2.94E+05	7.68E+05	-2.39
Sulfuretin-6-O-glucoside	3.54E+05	7.46E+05	-3.35
**3,3',4',5,6,7,8-heptamethoxyflavone**	**2.37E+04**	**5.13E+04**	**-7.74**
4',5,8-Trihydroxyflavanone	8.12E+04	5.35E+05	-3.39
Bracteatin	4.16E+06	1.29E+07	-3.39
Hispidulin 4'-glucoside	1.01E+05	7.40E+06	-3.16
3,4,5,2',4',6'-Hexahydroxychalcone 2'-glucoside	1.64E+05	8.55E+06	-3.32
Isomonospermoside	8.36E+04	3.13E+05	-1.03
Genestein G1	1.02E+05	1.39E+06	-1.23

Continued

**Mallotusinin**	**1.68E+06**	**5.93E+06**	**-3.18**
Robinetin	3.33E+06	8.20E+06	-1.52
2',4,4',6'-Tetrahydroxychalcone 4'-O-glucoside	1.07E+05	5.63E+05	-1.54
Nervilifordin B	5.22E+04	5.31E+05	-1.87
Glucodistylin-6"-glucoside	3.09E+04	6.61E+06	-1.45
Chrysoeriol-5,7-di-O-glucoside	6.73E+03	7.04E+04	-5.36
Tectochrysin-5-O-glucoside	1.32E+04	1.39E+05	-3.30
1,3,7-trihydroxy-5,6-dimethoxyxanthen-9-one	4.13E+04	3.70E+05	-3.10
Isomollupentin 2"-O-glucoside	7.61E+03	7.58E+04	-2.72
Hydroxy isoliquiritigenin glucoside	1.43E+06	2.92E+06	-1.64
Isocoreopsin	2.08E+05	4.89E+05	-6.19
1,3,6,8-tetrahydroxy-2,5-dimethoxyxanthen-9-one	1.41E+04	1.27E+05	-5.70
2',3',4',5,7-pentahydroxyflavone	1.10E+06	3.17E+06	-1.90
7,4'-Di-O-galloyltricetiflavan	4.09E+05	1.19E+06	-3.76
Arecatannin C1	2.01E+04	7.34E+04	-1.82
7,3'-Di-O-gallyoltricetiflavan	4.39E+05	1.20E+06	-1.30
Tricetin 5-(2"-dihydroxybenzoyl) hydroxyterephthalic acid	8.57E+03	3.53E+05	-2.39
1,3,6-trihydroxy-2,5,7-trimethoxyxanthen-9-one	3.80E+03	3.75E+04	-3.35
Methyl chebulagic acid	1.25E+03	1.07E+04	-7.74
**[2-(3,4-dihydroxyphenyl)-3,5,7-trihydroxy-3,4-dihydro-2h-1-benzopyran-4-yl]- -(4-hydroxyphenyl)-3,4-dihydro-2h-1-benzopyran-3,5,7-triol**	**1.84E+05**	**1.29E+04**	**3.83**
3,4-dihydroxyphenyl)-3,6-dihydroxy-7-[(2S,3R,4S,5S,6R)-3,4,5-trihydroxy- - (hydroxymethyl) oxan-2-yl] oxychromen-4-one	2.86E+06	7.96E+05	1.85
[6-[5,7-dihydroxy-2-(4-hydroxyphenyl)-4-oxochromen-3-yl]oxy-3,4,5- [7-trihydroxyoxan-2-yl]methyl 6-oxopyran-3-carboxylate	4.21E+06	6.43E+05	2.71
2alpha-(3,4,5-Trihydroxyphenyl)-4beta-(2,4,6-trihydroxyphenyl) chroman-3alpha,5,7-triol	6.68E+06	2.97E+05	4.49
hydroxy-8-methoxy-2-(2-methoxyphenyl)-7-{[(2s,3r,4s,5s,6r)-3,4,5-trihydroxy-6-(hydroxymethyl)oxan-2-yl]oxy}chromen-4-one	1.16E+05	4.32E+04	1.42
3-{[3,4-dihydroxy-6-(hydroxymethyl)-5-[(3,4,5-trihydroxy-6-{[(3,4,5-trihydroxy-6-methyloxan-2-yl)oxy]methyl}oxan-2-yl)oxy]oxan-2-yl]oxy}-5,7-dihydroxy-2-(4- hydroxyphenyl)-4H-chromen-4-one	4.90E+05	4.84E+04	3.34
3-[3,4-Dihydroxy-6-methyl-5-(3,4,5-trihydroxyoxan-2-yl)oxyoxan-2-yl] oxy-2-(3,4-dihydroxyphenyl)-5-hydroxychromen-4-one	1.01E+06	1.04E+05	3.28
[2-[5,7-dihydroxy-2-(4-hydroxyphenyl)-4-oxochromen-3-yl]oxy-4,5- [3-dihydroxyoxan-3-yl]3,4,5-trihydroxybenzoate	1.95E+06	1.99E+05	3.29

Continued

**3-{[(2R,3S,4R,5S,6S)-6-({[(2S,3R,4S)-3,4-dihydroxy-4-(hydroxymethyl) oxolan-2-yl]oxy}methyl)-3,4,5-trihydroxyoxan-2-yl]oxy}-5,7-dihydroxy-2-(4-hydroxyphenyl) chromen-4-one**	**2.13E+07**	**3.30E+06**	**2.69**
3-[(2R,3S,4S,5S)-3,4-dihydroxy-5-[(2S,3R,4S,5S,6R)-3,4,5-trihydroxy-6-(hydroxymethyl) oxan-2-yl]oxyoxan-2-yl] oxy-5,7-dihydroxy-2-(4-hydroxyphenyl) chromen-4-one	2.09E+07	4.62E+06	2.18
8-[(2S,3R,4S,5S,6R)-4,5-dihydroxy-6-(hydroxymethyl)-3-[(2S,3R,4S,5R)-3,4,5-trihydroxyoxan-2-yl]oxyoxan-2-yl] oxy-5,7-dihydroxy-2-(4-hydroxyphenyl) chromen-4-one	2.01E+07	4.76E+06	2.08
3-{[3,4-dihydroxy-6-(hydroxymethyl)-5-[(3,4,5-trihydroxy-6-methyloxan-2-yl) 4-oxy]oxan-2-yl]oxy}-5-hydroxy-2-(4-hydroxyphenyl)-7-[(3,4,5-trihydroxy -6-methyloxan-2-yl) oxy]-4H-chromen-4-one	1.67E+05	1.40E+04	3.58
(Z)-4,6-Dihydroxy-2-(4-Hydroxy-3-Methoxybenzylidene)-7-Methylbenzofuran-3 (2h)-One	6.51E+04	6.56E+03	3.31
2-(2,4-dihydroxyphenyl)-7-hydroxy-3-methoxy-5-[3,4,5-trihydroxy-6-(hydroxymethyl) oxan-2-yl] oxychromen-4-one	9.55E+04	5.72E+03	4.06
3,5-Dihydroxy-2-[3-methoxy-4-[3,4,5-trihydroxy-6-(hydroxymethyl) oxan-2-yl] oxyphenyl] chromen-4-one	1.17E+05	7.01E+06	-5.91
7-methoxy-3-(4-methoxyphenyl)-8-[(2S,3R,4S,5S,6R)-3,4,5-trihydroxy-6-(hydroxymethyl) oxan-2-yl] oxychromen-4-one	1.34E+05	5.04E+05	-1.92
(2R)-7-hydroxy-2-(4-hydroxyphenyl)-5-[(2S,3R,4S,5S,6R)-3,4,5-trihydroxy-6-[[(2R,3R,4S,5S,6R)-3,4,5-trihydroxy-6-(hydroxymethyl) oxan-2-yl] oxymethyl] oxan-2-yl] oxy-2,3-dihydrochromen-4-one	2.09E+05	1.88E+06	-3.17
3,8-dihydroxy-4,5-dimethoxy-1-[(2S,3R,4S,5S,6R)-3,4,5-trihydroxy-6-(hydroxymethyl) oxan-2-yl] oxyxanthen-9-one	2.57E+05	1.28E+06	-2.32
(2S)-2-(3,5-dihydroxyphenyl)-5,6-dihydroxy-7-[(2S,3R,4S,5S,6R)-3,4,5-trihydroxy-6-(hydroxymethyl) oxan-2-yl] oxy-2,3-dihydrochromen-4-one	2.86E+05	1.43E+06	-2.32
(3r,4r,6s)-6-{[(2r,3s,4s,5r,6s)-6-{[2-(3,4-dihydroxyphenyl)-5-hydroxy-4-oxochromen-7-yl]oxy}-3,4,5-trihydroxyoxan-2-yl]methoxy}-3,4,5-trihydroxyoxane-2- carboxylic acid	6.30E+04	5.36E+05	-3.09
2-(3-Methoxy-4-hydroxyphenyl)-6-[3-O-(beta-D-glucopyranosyl)-beta-D-glucopyranosyl]-5,7-dihydroxy-4H-1-benzopyran-4-one	9.67E+03	7.88E+04	-3.03
2-(3,4-dihydroxyphenyl)-5,6-dihydroxy-8-[(2S,3R,4R,5S,6R)-3,4,5-trihydroxy-6- (hydroxymethyl)oxan-2-yl]chromen-4-one	1.24E+04	1.26E+05	-3.34
1,8-dihydroxy-2,6-dimethoxy-5-{[(2s,3r,4s,5s,6r)-3,4,5-trihydroxy-6-(hydroxymethyl)oxan-2-yl]oxy}xanthen-9-one	1.09E+05	1.30E+06	-3.58

Table S3. Overview of transcriptome sequencing data acquired on the MGI sequencing platform.

Sample	Raw Reads	Clean Reads	Clean Base (G)	Error Rate (%)	Q20 (%)	Q30 (%)	GC (%)
AR-1	57447344	56129112	8.42	0.02	98.07	94.84	45.98
AR-2	54408178	53055964	7.96	0.02	98.25	95.33	45.24
AR-3	61310390	59290508	8.89	0.02	98.07	94.86	45.75
SR-1	63877822	62333562	9.35	0.02	98.12	94.99	45.79
SR-2	66460384	64741442	9.71	0.02	98.89	96.58	45.84
SR-3	58601776	57263580	8.59	0.02	98.01	94.69	45.6

Table S4. Number of reads of six samples mapped to reference sequences.

Sample	Total Reads	Total_Mapped (%)	Unique_Mapped (%)	Multiple_Mapped (%)
AR-1	56129112	50077798 (89.22%)	48180838 (85.84%)	1896960 (3.38%)
AR-2	53055964	47420546 (89.38%)	45491903 (85.74%)	1928643 (3.64%)
AR-3	59290508	52616224 (88.74%)	50567034 (85.29%)	2049190 (3.46%)
SR-1	62333562	55615161 (89.22%)	53441705 (85.74%)	2173456 (3.49%)
SR-2	64741442	58470303 (90.31%)	56163913 (86.75%)	2306390 (3.56%)
SR-3	57263580	50844733 (88.79%)	48883847 (85.37%)	1960886 (3.42%)

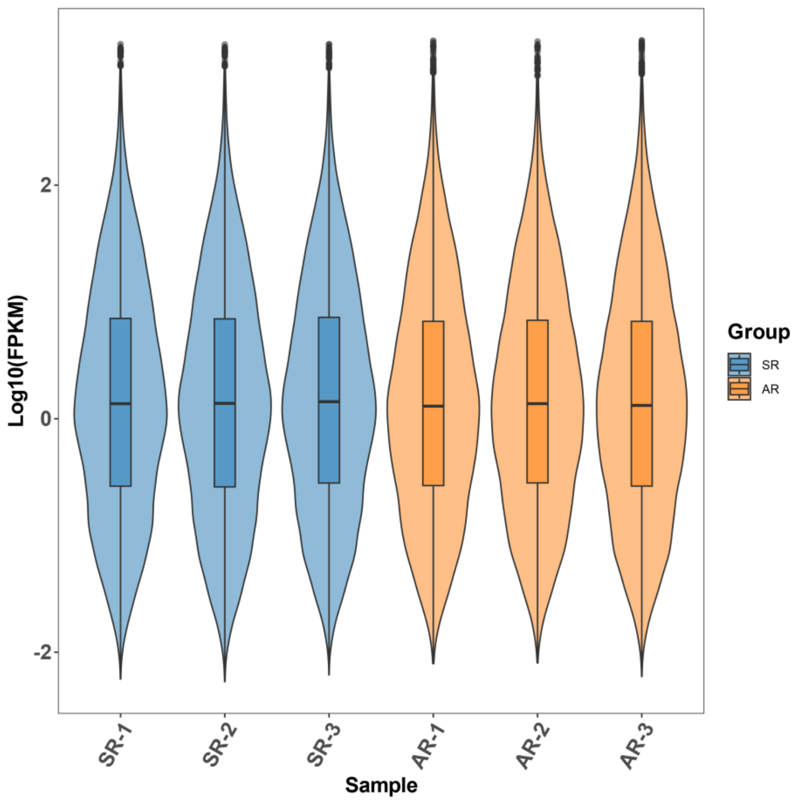

Figure S1. Distribution pattern of gene expression in the samples based on FPKM data.

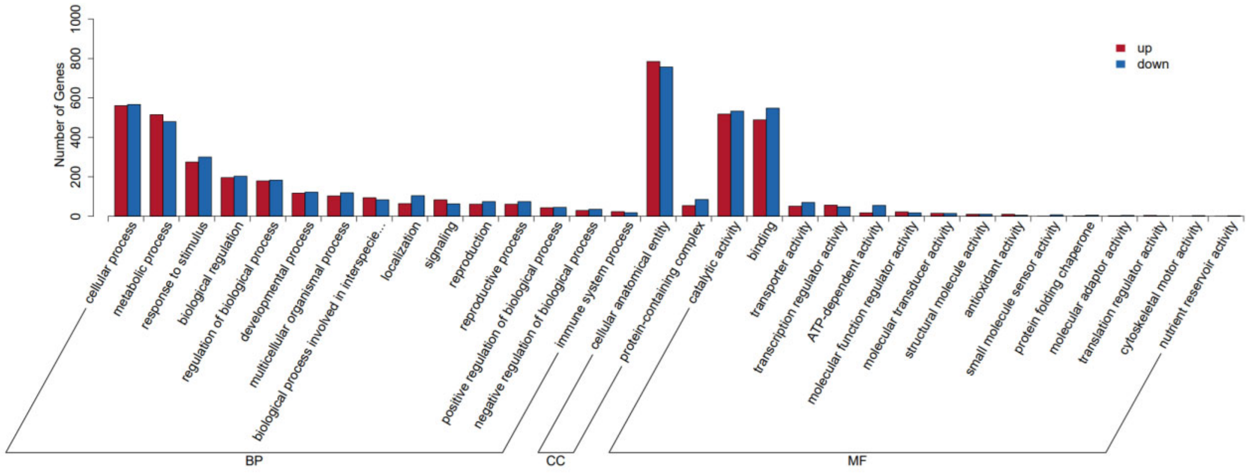

Figure S2. GO classification of annotated up- and down-regulated DEGs in AR vs. SR.

Table S5. FPKM values of DEGs related to the biosynthesis of flavonoids and phenylpropanoids between AR and SR.

Function	Gene ID	AR_Mean	SR_Mean	log2(FC)
Phenylpropanoid biosynthesis	RcHm_v2.0_Chr1g0317591	0.68	1.79	-1.33
	RcHm_v2.0_Chr1g0317701	9.12	27.95	-1.54
	RcHm_v2.0_Chr1g0339261	535.03	1257.07	-1.16
	RcHm_v2.0_Chr1g0365331	2.82	1.26	1.24
	RcHm_v2.0_Chr1g0367691	2.40	0.39	2.69
	RcHm_v2.0_Chr1g0371621	33.65	13.11	1.43
	RcHm_v2.0_Chr1g0382961	146.07	64.41	1.26
	RcHm_v2.0_Chr2g0092641	12.85	3.97	1.78
	RcHm_v2.0_Chr2g0107511	1.84	0.46	2.09
	RcHm_v2.0_Chr2g0110601	3.22	8.59	-1.34
	RcHm_v2.0_Chr2g0119681	1.74	4.26	-1.23
	RcHm_v2.0_Chr2g0132651	45.44	13.64	1.81
	RcHm_v2.0_Chr2g0142921	1.73	13.67	-2.90
	RcHm_v2.0_Chr2g0170391	22.64	10.39	1.20
	RcHm_v2.0_Chr3g0461001	105.08	36.80	1.59
	RcHm_v2.0_Chr3g0495951	2.70	1.36	1.06
	RcHm_v2.0_Chr4g0413701	3.44	11.03	-1.60
	RcHm_v2.0_Chr4g0431881	2.30	0.52	2.22
	RcHm_v2.0_Chr5g0017641	9.89	3.55	1.55
	RcHm_v2.0_Chr5g0017651	8.05	3.42	1.31
	RcHm_v2.0_Chr5g0017981	1.03	0.50	1.11
	RcHm_v2.0_Chr5g0066631	29.59	3.15	3.31
	RcHm_v2.0_Chr5g0072371	1.40	0.46	1.66
	RcHm_v2.0_Chr6g0295471	0.34	1.90	-2.44
	RcHm_v2.0_Chr6g0296031	3.49	1.11	1.72
	RcHm_v2.0_Chr7g0204981	12.96	31.32	-1.20
	RcHm_v2.0_Chr7g0209281	1.15	2.92	-1.28
	RcHm_v2.0_Chr7g0216761	1.20	3.80	-1.59
	RcHm_v2.0_Chr7g0216771	0.96	2.13	-1.07
Anthocyanin biosynthesis	RcHm_v2.0_Chr1g0316441	23.43	56.58	-1.19
	RcHm_v2.0_Chr1g0316451	9.44	29.32	-1.56
	RcHm_v2.0_Chr2g0153231	3.27	8.41	-1.29
	RcHm_v2.0_Chr7g0199941	24.89	58.03	-1.14
Flavone and flavonol biosynthesis	RcHm_v2.0_Chr1g0358281	3.04	0.99	1.69
	RcHm_v2.0_Chr1g0358291	0.99	0.24	2.14
	RcHm_v2.0_Chr2g0110741	51.74	16.33	1.74
	RcHm_v2.0_Chr4g0393121	16.02	5.39	1.65
Isoflavonoid biosynthesis	RcHm_v2.0_Chr2g0119541	0.43	0.99	-1.11
	RcHm_v2.0_Chr6g0308891	1.58	6.19	-1.90
	RcHm_v2.0_Chr6g0309031	1.03	0.47	1.20

Table S6. FPKM values of differentially expressed transcription factors between AR and SR.

Family	Gene ID	AR_Mean	SR_Mean	log2(FC)	Padj
	RcHm_v2.0_Chr1g0360311	2.07	97.68	-5.48	0.00E+00
	RcHm_v2.0_Chr2g0094411	2.19	1.09	1.10	6.60E-03
	RcHm_v2.0_Chr2g0094451	1.48	0.13	3.57	8.04E-11
	RcHm_v2.0_Chr2g0168581	2.06	5.08	-1.23	8.45E-15
	RcHm_v2.0_Chr3g0458721	1.55	3.40	-1.06	1.87E-08
	RcHm_v2.0_Chr4g0424331	0.21	1.13	-2.37	1.85E-07
	RcHm_v2.0_Chr5g0006031	0.85	0.42	1.07	2.08E-02
MYB	RcHm_v2.0_Chr5g0039751	9.90	3.88	1.43	1.28E-31
	RcHm_v2.0_Chr5g0040211	2.46	0.71	1.87	1.43E-10
	RcHm_v2.0_Chr6g0304811	1.89	0.43	2.22	1.64E-09
	RcHm_v2.0_Chr6g0307001	2.04	0.60	1.83	3.77E-09
	RcHm_v2.0_Chr6g0308731	202.81	94.13	1.18	9.88E-55
	RcHm_v2.0_Chr7g0177631	17.14	8.79	1.04	2.32E-15
	RcHm_v2.0_Chr7g0192781	0.92	2.32	-1.26	6.64E-06
	RcHm_v2.0_Chr7g0202031	1.02	0.45	1.26	1.73E-03
MYB-related	RcHm_v2.0_Chr2g0093071	2.71	0.80	1.84	6.56E-13
	RcHm_v2.0_Chr1g0348781	3.25	0.93	1.89	5.01E-17
	RcHm_v2.0_Chr1g0361191	7.19	3.42	1.15	1.60E-22
	RcHm_v2.0_Chr2g0126861	0.84	2.26	-1.37	8.73E-05
	RcHm_v2.0_Chr3g0480621	1.53	3.28	-1.03	5.93E-08
	RcHm_v2.0_Chr4g0399211	2.10	0.68	1.71	1.40E-06
bHLH	RcHm_v2.0_Chr4g0437041	1.59	4.50	-1.42	2.36E-25
	RcHm_v2.0_Chr5g0004791	1.71	0.82	1.14	2.78E-03
	RcHm_v2.0_Chr4g0440111	14.21	31.36	-1.07	1.04E-26
	RcHm_v2.0_Chr7g0185551	5.58	1.94	1.60	9.61E-16
	RcHm_v2.0_Chr7g0183781	11.28	5.82	1.03	1.67E-22
	RcHm_v2.0_Chr3g0451111	5.85	2.41	1.36	6.15E-21
	RcHm_v2.0_Chr1g0350911	0.90	1.89	-1.01	1.73E-04
	RcHm_v2.0_Chr1g0361611	0.40	1.25	-1.59	5.08E-04
	RcHm_v2.0_Chr1g0361631	0.73	1.90	-1.31	1.92E-04
NAC	RcHm_v2.0_Chr5g0026231	2.36	4.99	-1.01	4.23E-06
	RcHm_v2.0_Chr5g0034761	75.96	36.77	1.12	4.83E-43
	RcHm_v2.0_Chr5g0045961	19.66	5.78	1.84	8.15E-52
	RcHm_v2.0_Chr7g0230611	7.11	1.97	1.93	1.24E-16
	RcHm_v2.0_Chr1g0372431	0.81	1.89	-1.15	2.78E-04
	RcHm_v2.0_Chr3g0485711	0.71	0.28	1.43	3.13E-03
	RcHm_v2.0_Chr3g0487201	0.41	0.97	-1.18	1.23E-02
WRKY	RcHm_v2.0_Chr5g0011581	9.80	4.93	1.07	3.99E-11
	RcHm_v2.0_Chr5g0083891	0.93	2.69	-1.46	1.76E-04
	RcHm_v2.0_Chr7g0180741	1.13	3.27	-1.46	2.79E-13
	RcHm_v2.0_Chr7g0202671	16.90	5.38	1.72	2.55E-40

Continued

	RcHm_v2.0_Chr2g0125901	0.90	0.41	1.21	2.18E-02
	RcHm_v2.0_Chr2g0128051	3.68	20.84	-2.42	1.20E-60
	RcHm_v2.0_Chr2g0154791	4.02	2.04	1.05	2.56E-04
	RcHm_v2.0_Chr2g0166751	5.91	2.07	1.59	6.59E-10
HB-WOX	RcHm_v2.0_Chr3g0461511	1.03	0.25	2.13	5.38E-05
	RcHm_v2.0_Chr3g0467561	3.31	1.09	1.68	3.05E-06
	RcHm_v2.0_Chr7g0180191	7.25	3.76	1.02	2.40E-06
	RcHm_v2.0_Chr7g0186381	0.24	2.04	-3.01	2.07E-06
	RcHm_v2.0_Chr7g0201031	0.60	3.92	-2.63	3.56E-10
HB-HD-ZIP	RcHm_v2.0_Chr4g0398491	2.05	0.74	1.55	8.09E-05
HB-other	RcHm_v2.0_Chr2g0148261	0.64	1.34	-1.00	1.63E-07
	RcHm_v2.0_Chr1g0371151	3.77	9.43	-1.24	4.79E-20
	RcHm_v2.0_Chr2g0085851	1.05	2.95	-1.41	1.36E-04
	RcHm_v2.0_Chr5g0032721	37.23	16.07	1.29	1.06E-07
AP2/ERF	RcHm_v2.0_Chr7g0204641	3.64	1.67	1.21	6.21E-09
	RcHm_v2.0_Chr7g0231481	3.30	9.46	-1.44	9.98E-14
	RcHm_v2.0_Chr7g0231501	7.73	22.81	-1.48	8.70E-26
	RcHm_v2.0_Chr1g0318281	3.54	1.53	1.28	1.20E-09
MADS-MIKC	RcHm_v2.0_Chr7g0236811	0.49	1.27	-1.31	3.10E-06
	RcHm_v2.0_Chr5g0012431	8.69	0.52	4.13	1.26E-65
MADS-M-type	RcHm_v2.0_Chr3g0477451	2.51	6.09	-1.20	3.35E-06
	RcHm_v2.0_Chr1g0366121	2.82	1.33	1.16	5.71E-04
	RcHm_v2.0_Chr3g0467531	0.52	1.70	-1.62	7.55E-04
	RcHm_v2.0_Chr3g0467541	0.91	3.01	-1.63	2.19E-04
C2H2	RcHm_v2.0_Chr5g0062951	15.55	8.07	1.03	1.07E-11
	RcHm_v2.0_Chr5g0062991	9.28	2.80	1.80	6.28E-16
	RcHm_v2.0_Chr6g0309391	332.54	170.51	1.04	4.99E-10
	RcHm_v2.0_Chr6g0311101	7.95	3.74	1.17	2.52E-21
C3H	RcHm_v2.0_Chr2g0099611	39.47	20.53	1.02	2.35E-42
	RcHm_v2.0_Chr3g0468431	27.00	58.89	-1.05	1.02E-43
B3	RcHm_v2.0_Chr3g0474911	2.95	1.18	1.39	3.41E-12
	RcHm_v2.0_Chr6g0259881	2.35	1.12	1.15	3.69E-06
B3-ARF	RcHm_v2.0_Chr6g0292551	2.38	1.14	1.14	1.69E-09
	RcHm_v2.0_Chr5g0065621	3.93	1.18	1.82	4.80E-21
GRAS	RcHm_v2.0_Chr5g0065811	16.58	4.97	1.82	5.36E-81
	RcHm_v2.0_Chr3g0493011	13.53	32.50	-1.19	3.10E-47
C2C2-GATA	RcHm_v2.0_Chr2g0093271	7.12	2.62	1.52	5.41E-23
	RcHm_v2.0_Chr6g0310911	3.26	1.39	1.31	3.01E-07
C2C2-CO-like	RcHm_v2.0_Chr4g0429371	7.77	19.19	-1.23	3.89E-52
bZIP	RcHm_v2.0_Chr1g0318951	0.87	0.31	1.57	1.18E-03
	RcHm_v2.0_Chr4g0436911	29.51	14.62	1.09	7.33E-24
SBP	RcHm_v2.0_Chr4g0437871	19.49	49.41	-1.27	2.02E-56
	RcHm_v2.0_Chr5g0032861	0.31	1.03	-1.66	4.14E-04

Continued

FAR1	novel.2377	29.34	12.78	1.27	1.08E-16
	RcHm_v2.0_Chr2g0118731	0.53	1.50	-1.42	4.48E-09
MED7	RcHm_v2.0_Chr3g0478001	0.51	2.19	-2.03	1.34E-05
MED6	RcHm_v2.0_Chr5g0064381	6.09	1.20	2.43	2.26E-08
Jumonji	RcHm_v2.0_Chr1g0346441	0.41	0.97	-1.18	2.99E-05
HMG	RcHm_v2.0_Chr1g0370021	7.41	20.71	-1.41	9.06E-35
Alfin-like	RcHm_v2.0_Chr1g0370381	2.82	9.51	-1.67	1.55E-24
Pseudo ARR-B	RcHm_v2.0_Chr1g0382871	0.87	4.88	-2.42	2.76E-44
Tify	RcHm_v2.0_Chr2g0096551	20.18	8.54	1.32	2.11E-40
TRAF	RcHm_v2.0_Chr2g0124891	1.18	2.95	-1.24	8.75E-12
Trihelix	RcHm_v2.0_Chr3g0471711	0.48	1.15	-1.20	8.20E-05
HSF	RcHm_v2.0_Chr3g0472131	2.93	16.51	-2.42	2.60E-37
CPP	RcHm_v2.0_Chr4g0387371	0.20	1.75	-3.05	1.90E-24
GNAT	RcHm_v2.0_Chr4g0415691	0.86	2.96	-1.70	1.78E-05
TCP	RcHm_v2.0_Chr4g0440011	10.17	4.82	1.15	1.29E-25
Others	RcHm_v2.0_Chr5g0005681	56.88	29.99	1.00	1.09E-33
LOB	RcHm_v2.0_Chr5g0019231	0.33	2.21	-2.67	2.93E-12
PLATZ	RcHm_v2.0_Chr5g0056371	6.69	3.24	1.12	2.35E-12
LUG	RcHm_v2.0_Chr6g0312941	10.38	24.89	-1.19	6.22E-56
TAZ	RcHm_v2.0_Chr7g0191831	169.35	66.90	1.42	2.00E-103
SNF-BAF60b	RcHm_v2.0_Chr7g0236391	7.75	1.38	2.56	2.23E-50

Table S7. FPKM values of differentially expressed *GSTs*, *ABCCs*, and *MATEs* between AR and SR.

Family	Gene ID	AR_Mean	SR_Mean	log2(FC)
GST	RcHm_v2.0_Chr3g0464521	1.45	3.39	-1.16
	RcHm_v2.0_Chr3g0468161	8.47	21.23	-1.25
	RcHm_v2.0_Chr5g0027341	3.93	8.68	-1.07
	RcHm_v2.0_Chr6g0285761	1.85	4.95	-1.35
ABCC	RcHm_v2.0_Chr7g0215041	0.32	3.68	-3.46
	RcHm_v2.0_Chr2g0142771	10.73	26.58	-1.24
	RcHm_v2.0_Chr2g0142761	0.90	5.03	-2.41
	RcHm_v2.0_Chr0c20g0500281	0.25	0.93	-1.81
	RcHm_v2.0_Chr4g0406401	0.45	1.02	-1.11
MATE	RcHm_v2.0_Chr2g0140591	0.55	1.64	-1.50
	RcHm_v2.0_Chr7g0199211	0.23	0.94	-1.93
	RcHm_v2.0_Chr5g0052011	4.07	10.14	-1.24
	RcHm_v2.0_Chr3g0481351	0.42	1.38	-1.65
	RcHm_v2.0_Chr4g0433941	0.74	1.62	-1.07
	RcHm_v2.0_Chr4g0423121	0.54	1.47	-1.37
	RcHm_v2.0_Chr4g0432901	2.81	7.07	-1.26
	RcHm_v2.0_Chr4g0433941	0.74	1.62	-1.07
	RcHm_v2.0_Chr7g0185081	20.14	4.25	2.32
	RcHm_v2.0_Chr2g0159351	12.26	4.87	1.41
	novel.1949	4.05	1.69	1.34
	RcHm_v2.0_Chr5g0003331	1.46	0.43	1.85

Table S8. FPKM values of structural genes related to flavonoid biosynthesis between AR and SR.

Genes	Genes ID	SR_Mean	AR_Mean	log2(FC)
PAL	RcHm_v2.0_Chr1g0363011	31.27	38.39	0.37
	RcHm_v2.0_Chr3g0469861	42.74	58.27	0.52
	RcHm_v2.0_Chr7g0212181	1.24	1.38	0.23
C4H	RcHm_v2.0_Chr5g0073351	144.03	135.49	-0.01
4CL	RcHm_v2.0_Chr1g0384231	90.39	97.56	0.19
	RcHm_v2.0_Chr4g0430951	1.07	0.92	-0.15
	RcHm_v2.0_Chr5g0057621	3.24	3.48	0.17
	RcHm_v2.0_Chr5g0057631	29.41	40.42	0.53
	RcHm_v2.0_Chr4g0402711	10.78	6.85	-0.58
CHS	RcHm_v2.0_Chr1g0316441	56.58	23.43	-1.19
	RcHm_v2.0_Chr1g0316451	29.32	9.44	-1.56
	RcHm_v2.0_Chr1g0316461	60.01	38.28	-0.57
CHI	RcHm_v2.0_Chr1g0372181	9.23	5.43	-0.69
	RcHm_v2.0_Chr1g0365111	30.10	31.24	0.13
F3H	RcHm_v2.0_Chr2g0099421	227.19	149.38	-0.53
F3’H	RcHm_v2.0_Chr7g0182961	1.92	1.51	-0.27
	RcHm_v2.0_Chr7g0202001	15.14	13.41	-0.10
FLS	RcHm_v2.0_Chr6g0295121	21.73	25.11	0.28
DFR	RcHm_v2.0_Chr6g0301421	37.32	29.84	-0.25
ANS	RcHm_v2.0_Chr7g0199941	58.03	24.89	-1.14
	RcHm_v2.0_Chr2g0152971	3.52	2.23	-0.58
ANR	RcHm_v2.0_Chr5g0004611	4.26	5.72	0.50
	RcHm_v2.0_Chr7g0195261	59.44	77.40	0.46
LAR	RcHm_v2.0_Chr4g0435881	63.59	49.10	-0.30
5,3-GT	RcHm_v2.0_Chr5g0046041	1.57	2.33	0.64
	RcHm_v2.0_Chr1g0378941	7.88	8.88	0.25
3-GT	RcHm_v2.0_Chr6g0302721	3.95	5.24	0.48
	RcHm_v2.0_Chr2g0153231	8.41	3.27	-1.29
	RcHm_v2.0_Chr1g0383951	2.89	5.24	0.93
COMT	RcHm_v2.0_Chr1g0367691	0.39	2.40	2.69
	RcHm_v2.0_Chr1g0382961	64.41	146.07	1.26
	RcHm_v2.0_Chr3g0471211	1.49	1.21	-0.21
	RcHm_v2.0_Chr4g0396271	32.27	61.11	0.99
	RcHm_v2.0_Chr6g0296031	1.11	3.49	1.72
CCoAOMT	RcHm_v2.0_Chr2g0092641	3.97	12.85	1.78
	RcHm_v2.0_Chr6g0256691	5.60	8.09	0.60
	RcHm_v2.0_Chr2g0092661	4.38	4.05	-0.04
	RcHm_v2.0_Chr2g0092671	32.95	32.92	0.07

Table S9. Primer sequences of genes used for qRT-PCR verification

Gene	primer sequence
RchiOBHmChr1g0316451	5' AACCTTTGAACATCACGGACTG 3'<br>5' CACCCCACTCCAAGCCCT 3'
RchiOBHmChr7g0199941	5' GCCTCAAACACCTTCCGATTA 3'<br>5' GACCTCCTTCTCCAGCCTCC 3'
RchiOBHmChr1g0360311	5' AGAAGAAGCAGAACCACAAAGC 3'<br>5' GAGAACACCACCCCACCCT 3'
RchiOBHmChr4g0436911	5' CAATGGAGCATCAGTCTTCAGC 3'<br>5' TCATCCAAGTGCTTCTGTTTCC 3'
RchiOBHmChr5g0034761	5' TCCGAGCAAAGAGCAGAGC 3'<br>5' GACCCGAGATTCCCCAGAT 3'
RchiOBHmChr7g0231501	5' TGAAGGGAGTGCGTCAAAGAA 3'<br>5' CGGCAAGATTGTGGGAAAGT 3'
RchiOBHmChr7g0202671	5' ACCAGGATGAAATGATGGACAG 3'<br>5' CTATATTGCTAGGAAGGACCACACT 3'
RchiOBHmChr1g0382961	5' TACGGTTTGGGACCTGTGTGT 3'<br>5' GGTAATGGTGGAGTGGTCAGC 3'
RcACTIN	5' TGTCTGTGATAATGGGACTGGA 3'<br>5' ACAATACTTGGGAACACGGCTC 3'

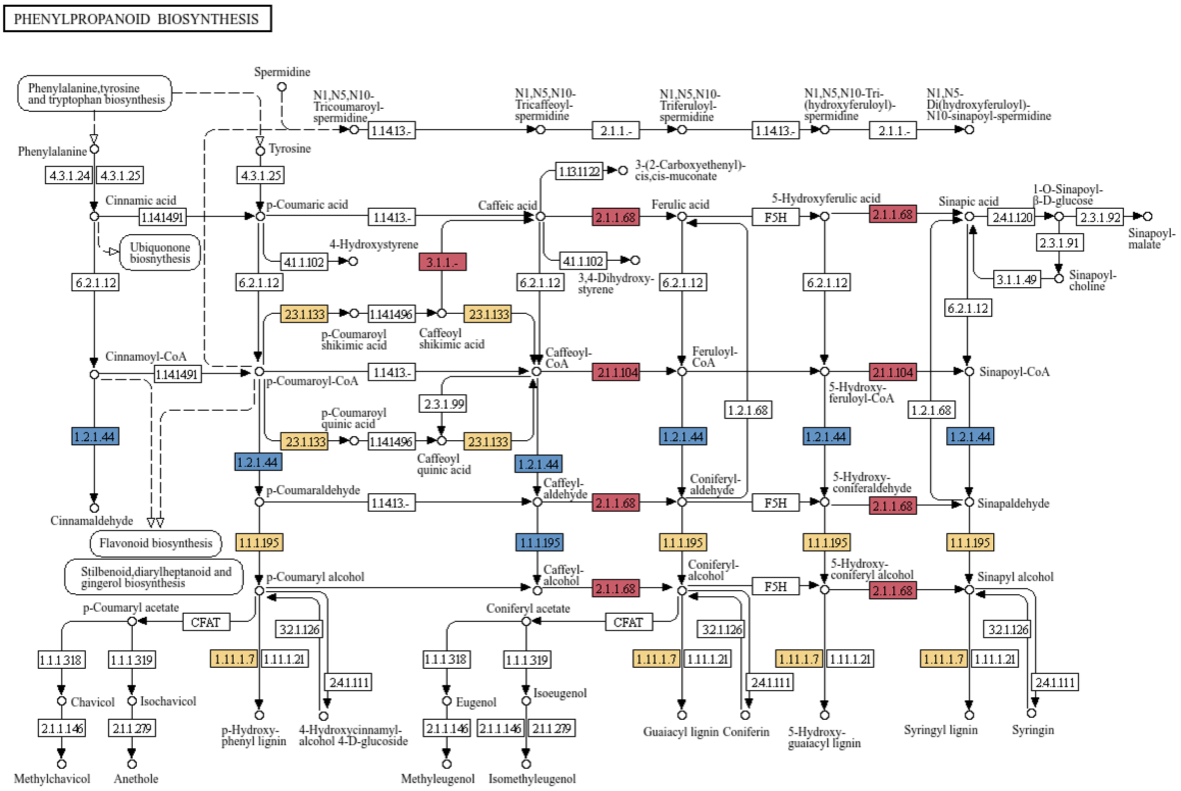

Figure S3. Biosynthetic pathway of phenylpropanoids in AR vs. SR. Red rectangles represent up-regulated gene expression. Blue rectangles represent down-regulated gene expression. Orange rectangles represent up-regulated and down-regulated gene expression.

Table S10. FPKM values of DEGs for network analysis between AR and SR.

Gene	Gene ID	SR-1	SR-2	SR-3	AR-1	AR-2	AR-3
CHS	Chr1g0316441	58.4864	57.2131	54.055	25.7015	20.4515	24.1465
CHS	Chr1g0316451	26.2646	32.3162	29.3726	10.7218	9.1316	8.48
ANS	Chr7g0199941	60.3123	58.3505	55.4308	25.5629	22.9101	26.2077
3GT	Chr2g0153231	8.6719	7.9816	8.5687	3.2773	3.3549	3.1752
CCoAOMT	Chr2g0092641	3.9714	3.4331	4.4965	12.5677	13.1685	12.812
COMT	Chr1g0367691	0.4424	0.4239	0.3017	2.1134	2.2173	2.864
COMT	Chr1g0382961	65.6412	64.0207	63.5596	150.9617	139.3474	147.9078
ABCC	Chr2g0142761	5.2937	5.277	4.5053	0.8865	0.8971	0.9129
ABCC	Chr2g0142771	26.8311	26.5435	26.3795	10.0729	12.213	9.8942
MATE	Chr5g0052011	9.6059	10.1903	10.6109	3.6276	4.7776	3.8181
MATE	Chr7g0185081	3.9751	4.5081	4.2786	20.4865	19.9607	19.9759
MATE	Chr2g0159351	4.7812	5.1404	4.6862	12.3161	11.749	12.7029
MATE	Chr2g0112071	129.7249	132.623	132.2695	227.9651	240.2065	239.7439
GST	Chr1g0364661	18.7197	16.2654	17.6826	5.0648	6.0661	4.6816
GST	Chr3g0468161	21.7217	22.6895	19.2811	9.4912	7.6371	8.2839
MYB	Chr6g0308731	93.53	93.95	94.91	220.29	184.22	203.93
MYB	Chr1g0360311	97.48	97.27	98.29	2.43	1.64	2.14
bHLH	Chr4g0440111	30.57	30.25	33.27	14.49	14.10	14.03
MADS	Chr5g0012431	0.52	0.60	0.44	8.57	9.23	8.2715

Table S11. DAMs for network analysis between AR and SR.

Compounds	Index	SR-1	SR-2	SR-3	AR-1	AR-2	AR-3
Cyanidin 3,5-O-diglucoside	Zbjp001957	6097052.189	7257879.08	8159590.061	244576.8298	254212.198	243096.4887
Pelargonin 3,5-O-diglucoside	Zbsp002083	557998.2505	760367.7497	578346.2022	80729.58175	78916.14343	80434.63516
Peonidin 3-O-sophoroside	Waptp02347	3482154.705	4250856.09	3282269.568	41128.55993	48346.33285	47103.87012
Peonidin 3-O-glucoside	Zblp002396	7689298.992	7553865.347	7141776.009	120841.4958	114513.3551	114879.5127
Cyanidin 3-O-beta-D-sambubioside	Zmjp001877	6882863.27	7694123.056	5421682.67	822681.5569	822681.5569	822681.5569
Cyanidin-3-O-galloyl-galactoside	Zasp002650	2290059.169	2549876.641	1701027.951	174361.6808	174361.6808	174361.6808
Cyanidin-3-diglucoside-5-glucoside	Zbzp001964	42153.87416	36822.27009	31065.22517	3600.187461	3600.187461	3600.187461
Cyanidin 3,3',5-tri-O-glucoside	Lmqp001551	98451.03497	104523.0368	98139.66937	13324.09927	13324.09927	13324.09927
Cyanidin-3-O-(6''-O-feruloyl)glucoside	Zmmp002642	570130.8452	584113.2627	584110.9296	61795.28689	61795.28689	61795.28689
Peonidin-3,5-O-diglucoside	Zbpp001841	3254728.229	3652473.033	3230171.207	48992.7778	47207.73895	38454.167
Cyanidin 3-O-(beta-D-xylosyl-(1->2)-beta-D-galactoside)	Lmjp001367	7268000.077	7874905.992	6565406.677	58925.26165	52350.60335	57247.24773
Quercetin	MWSHY0029	142386.08	227807.9119	148162.517	56051.79291	48210.10876	67797.48734
Taxifolin	mws0044	240659.9226	274221.0686	306091.3861	26018.59043	13004.71731	17661.54003
Kaempferol	MWSHY0051	42012.09346	61595.58151	36768.22722	838632.0216	924600.4837	955155.5803

Table S12. The correlation between transcripts and metabolites related to anthocyanins in rose petals.

Genes ID	Metabolites	Correlation	P-Value
Chr1g0316441	Zbjp001957	0.982082606	0.000478673
Chr1g0316441	Zbsp002083	0.984285778	0.000368465
Chr1g0316441	Waptp02347	0.98415069	0.00037481
Chr1g0316441	Zblp002396	0.988417575	0.000200452
Chr1g0316441	Zmjp001877	0.986465729	0.000273525
Chr1g0316441	Zasp002650	0.986766343	0.000261536
Chr1g0316441	Zbzp001964	0.988049199	0.000213379
Chr1g0316441	Lmqp001551	0.987101793	0.000248473
Chr1g0316441	Zmmp002642	0.986701443	0.000264102
Chr1g0316441	Zbpp001841	0.985922222	0.000295881
Chr1g0316441	Lmjp001367	0.989731269	0.000157629
Chr1g0316441	MWSHY0029	0.956625499	0.00278122
Chr1g0316441	mws0044	0.993638122	6.05815E-05
Chr1g0316441	MWSHY0051	-0.980890843	0.000544251
Chr1g0316451	Zbjp001957	0.989151331	0.000175902
Chr1g0316451	Zbsp002083	0.991881214	9.86045E-05
Chr1g0316451	Waptp02347	0.986486183	0.000272701
Chr1g0316451	Zblp002396	0.987757547	0.000223899
Chr1g0316451	Zmjp001877	0.985151132	0.000329096
Chr1g0316451	Zasp002650	0.984780115	0.000345705
Chr1g0316451	Zbzp001964	0.980518264	0.00056561
Chr1g0316451	Lmqp001551	0.988740363	0.000189455
Chr1g0316451	Zmmp002642	0.987855077	0.000220353
Chr1g0316451	Zbpp001841	0.991486731	0.000108405
Chr1g0316451	Lmjp001367	0.988538098	0.00019631
Chr1g0316451	MWSHY0029	0.953777881	0.00315535
Chr1g0316451	mws0044	0.991031487	0.000120291
Chr1g0316451	MWSHY0051	-0.977435512	0.00075799
Chr7g0199941	Zbjp001957	0.988717015	0.00019024
Chr7g0199941	Zbsp002083	0.98987873	0.000153142
Chr7g0199941	Waptp02347	0.992374945	8.69905E-05
Chr7g0199941	Zblp002396	0.994464804	4.58728E-05
Chr7g0199941	Zmjp001877	0.993066379	7.1946E-05
Chr7g0199941	Zasp002650	0.993416635	6.48684E-05
Chr7g0199941	Zbzp001964	0.995123974	3.56055E-05
Chr7g0199941	Lmqp001551	0.993623179	6.08661E-05
Chr7g0199941	Zmmp002642	0.993274324	6.77E-05
Chr7g0199941	Zbpp001841	0.991271984	0.000113935
Chr7g0199941	Lmjp001367	0.995411477	3.15335E-05
Chr7g0199941	MWSHY0029	0.959428068	0.00243573
Chr7g0199941	mws0044	0.988976849	0.000181595
Chr7g0199941	MWSHY0051	-0.986102971	0.000288349

Continued

Chr7g0199941	Zmmp002642	0.993274324	6.77E-05
Chr7g0199941	Zbpp001841	0.991271984	0.000113935
Chr7g0199941	Lmjp001367	0.995411477	3.15335E-05
Chr7g0199941	MWSHY0029	0.959428068	0.00243573
Chr7g0199941	mws0044	0.988976849	0.000181595
Chr7g0199941	MWSHY0051	-0.986102971	0.000288349
Chr2g0153231	Zbjp001957	0.996209424	2.15255E-05
Chr2g0153231	Zbsp002083	0.98809478	0.000211758
Chr2g0153231	Waptp02347	0.995258279	3.36726E-05
Chr2g0153231	Zblp002396	0.997757718	7.53611E-06
Chr2g0153231	Zmjp001877	0.989552391	0.000163159
Chr2g0153231	Zasp002650	0.990155302	0.0001449
Chr2g0153231	Zbzp001964	0.995320988	3.27885E-05
Chr2g0153231	Lmqp001551	0.996633318	1.69827E-05
Chr2g0153231	Zmmp002642	0.997664909	8.17261E-06
Chr2g0153231	Zbpp001841	0.997153591	1.21415E-05
Chr2g0153231	Lmjp001367	0.996376454	1.96713E-05
Chr2g0153231	MWSHY0029	0.927410816	0.007712541
Chr2g0153231	mws0044	0.983596761	0.000401393
Chr2g0153231	MWSHY0051	-0.997792534	7.30398E-06
Chr2g0092641	Zbjp001957	-0.987338151	0.000239469
Chr2g0092641	Zbsp002083	-0.997150629	1.21668E-05
Chr2g0092641	Waptp02347	-0.994298683	4.86649E-05
Chr2g0092641	Zblp002396	-0.992182167	9.14389E-05
Chr2g0092641	Zmjp001877	-0.998806199	2.13689E-06
Chr2g0092641	Zasp002650	-0.99852294	3.27095E-06
Chr2g0092641	Zbzp001964	-0.993414228	6.49158E-05
Chr2g0092641	Lmqp001551	-0.993346587	6.62546E-05
Chr2g0092641	Zmmp002642	-0.991156981	0.000116953
Chr2g0092641	Zbpp001841	-0.992596994	8.20039E-05
Chr2g0092641	Lmjp001367	-0.993944183	5.48983E-05
Chr2g0092641	MWSHY0029	-0.972364112	0.00113506
Chr2g0092641	mws0044	-0.979700504	0.000613922
Chr2g0092641	MWSHY0051	0.972785536	0.001100863
Chr1g0367691	Zbjp001957	-0.99091161	0.000123523
Chr1g0367691	Zbsp002083	-0.977666992	0.000742575
Chr1g0367691	Waptp02347	-0.981276643	0.000522564
Chr1g0367691	Zblp002396	-0.985126523	0.000330185
Chr1g0367691	Zmjp001877	-0.969482235	0.00138279
Chr1g0367691	Zasp002650	-0.969802785	0.00135404
Chr1g0367691	Zbzp001964	-0.973904468	0.00101258
Chr1g0367691	Lmqp001551	-0.984714161	0.0003487
Chr1g0367691	Zmmp002642	-0.986640932	0.000266505

Continued

Chr1g0367691	Zbpp001841	-0.987796624	0.000222475
Chr1g0367691	Lmjp001367	-0.98315898	0.000423042
Chr1g0367691	mws0044	-0.98331694	0.000415165
Chr1g0367691	MWSHY0051	0.991049363	0.000119812
Chr1g0382961	Zbjp001957	-0.998329551	4.18327E-06
Chr1g0382961	Zbsp002083	-0.994010732	5.36996E-05
Chr1g0382961	Waptp02347	-0.998245717	4.61357E-06
Chr1g0382961	Zblp002396	-0.99733602	1.06357E-05
Chr1g0382961	Zmjp001877	-0.992162333	9.19028E-05
Chr1g0382961	Zasp002650	-0.992339868	8.77917E-05
Chr1g0382961	Zbzp001964	-0.993416842	6.48643E-05
Chr1g0382961	Lmqp001551	-0.99791952	6.48809E-06
Chr1g0382961	Zmmp002642	-0.99814113	5.17988E-06
Chr1g0382961	Zbpp001841	-0.997605457	8.59389E-06
Chr1g0382961	Lmjp001367	-0.996655068	1.67641E-05
Chr1g0382961	MWSHY0029	-0.943216292	0.004745038
Chr1g0382961	mws0044	-0.979045532	0.000654034
Chr1g0382961	MWSHY0051	0.991446318	0.000109435
Chr2g0142761	Zbjp001957	0.994185479	5.06147E-05
Chr2g0142761	Zbsp002083	0.995816832	2.62117E-05
Chr2g0142761	Waptp02347	0.998766648	2.2808E-06
Chr2g0142761	Zblp002396	0.998527572	3.25047E-06
Chr2g0142761	Zmjp001877	0.9989298	1.71738E-06
Chr2g0142761	Zasp002650	0.999140603	1.10753E-06
Chr2g0142761	Zbzp001964	0.999398773	5.42101E-07
Chr2g0142761	Lmqp001551	0.998488858	3.4236E-06
Chr2g0142761	Zmmp002642	0.997781587	7.37657E-06
Chr2g0142761	Zbpp001841	0.997537169	9.09083E-06
Chr2g0142761	Lmjp001367	0.998869965	1.91475E-06
Chr2g0142761	MWSHY0029	0.955505085	0.002925651
Chr2g0142761	mws0044	0.982654567	0.000448687
Chr2g0142761	MWSHY0051	-0.987777377	0.000223176
Chr2g0142771	Zbjp001957	0.988587301	0.000194631
Chr2g0142771	Zbsp002083	0.983509609	0.000405657
Chr2g0142771	Waptp02347	0.990216328	0.000143112
Chr2g0142771	Zblp002396	0.988625705	0.000193326
Chr2g0142771	Zmjp001877	0.984947576	0.000338158
Chr2g0142771	Zasp002650	0.985247154	0.000324864
Chr2g0142771	Zbzp001964	0.987129431	0.000247411
Chr2g0142771	Lmqp001551	0.988972469	0.000181739
Chr2g0142771	Zmmp002642	0.989102156	0.000177497
Chr2g0142771	Zbpp001841	0.990661858	0.000130394
Chr2g0142771	Lmjp001367	0.986921913	0.000255436

Continued

Chr2g0142771	mws0044	0.959919108	0.002377522
Chr2g0142771	MWSHY0051	-0.982726188	0.000445
Chr5g0052011	Zbjp001957	0.984833867	0.000343273
Chr5g0052011	Zbsp002083	0.977147257	0.000777404
Chr5g0052011	Waptp02347	0.983539863	0.000404174
Chr5g0052011	Zblp002396	0.979828179	0.00060625
Chr5g0052011	Zmjp001877	0.973212485	0.001066745
Chr5g0052011	Zasp002650	0.973304847	0.001059435
Chr5g0052011	Zbzp001964	0.97408339	0.000998802
Chr5g0052011	Lmqp001551	0.981440937	0.000513462
Chr5g0052011	Zmmp002642	0.981946621	0.000485945
Chr5g0052011	Zbpp001841	0.982146388	0.000475282
Chr5g0052011	Lmjp001367	0.977959099	0.000723348
Chr5g0052011	mws0044	0.948623127	0.003891568
Chr5g0052011	MWSHY0051	-0.974849366	0.000940877
Chr7g0185081	Zbjp001957	-0.996000255	2.39649E-05
Chr7g0185081	Zbsp002083	-0.990435848	0.000136772
Chr7g0185081	Waptp02347	-0.997082077	1.2759E-05
Chr7g0185081	Zblp002396	-0.998820078	2.0875E-06
Chr7g0185081	Zmjp001877	-0.992957887	7.42124E-05
Chr7g0185081	Zasp002650	-0.99350985	6.30464E-05
Chr7g0185081	Zbzp001964	-0.997824424	7.09455E-06
Chr7g0185081	Lmqp001551	-0.997887787	6.68745E-06
Chr7g0185081	Zmmp002642	-0.998507806	3.3383E-06
Chr7g0185081	Zbpp001841	-0.997168761	1.20125E-05
Chr7g0185081	Lmjp001367	-0.99803063	5.81381E-06
Chr7g0185081	MWSHY0029	-0.937971507	0.005651973
Chr7g0185081	mws0044	-0.983849213	0.000389165
Chr7g0185081	MWSHY0051	0.996018129	2.37514E-05
Chr2g0159351	Zbjp001957	-0.996337907	2.00918E-05
Chr2g0159351	Zbsp002083	-0.98671875	0.000263416
Chr2g0159351	Waptp02347	-0.994197157	5.04118E-05
Chr2g0159351	Zblp002396	-0.996480639	1.85571E-05
Chr2g0159351	Zmjp001877	-0.986896266	0.000256437
Chr2g0159351	Zasp002650	-0.987485969	0.000233922
Chr2g0159351	Zbzp001964	-0.992814777	7.72557E-05
Chr2g0159351	Lmqp001551	-0.995533129	2.98848E-05
Chr2g0159351	Zmmp002642	-0.996791941	1.5421E-05
Chr2g0159351	Zbpp001841	-0.996286014	2.06649E-05
Chr2g0159351	Lmjp001367	-0.994818371	4.02044E-05
Chr2g0159351	MWSHY0029	-0.923049297	0.008654288
Chr2g0159351	mws0044	-0.981500948	0.000510157
Chr2g0159351	MWSHY0051	0.997713019	7.83944E-06

Continued

Chr2g0112071	Zbjp001957	-0.995463469	3.08235E-05
Chr2g0112071	Zbsp002083	-0.992246684	8.99378E-05
Chr2g0112071	Waptp02347	-0.99544104	3.11288E-05
Chr2g0112071	Zblp002396	-0.998554593	3.13229E-06
Chr2g0112071	Zmjp001877	-0.993354477	6.60977E-05
Chr2g0112071	Zasp002650	-0.993747932	5.85103E-05
Chr2g0112071	Zbzp001964	-0.996492944	1.84276E-05
Chr2g0112071	Lmqp001551	-0.997529082	9.15061E-06
Chr2g0112071	Zmmp002642	-0.99780683	7.20972E-06
Chr2g0112071	Zbpp001841	-0.998197018	4.87319E-06
Chr2g0112071	Lmjp001367	-0.998250681	4.5875E-06
Chr2g0112071	MWSHY0029	-0.943192813	0.004748925
Chr2g0112071	mws0044	-0.992758137	7.8477E-05
Chr2g0112071	MWSHY0051	0.994740209	4.14253E-05
Chr1g0364661	Zbjp001957	0.987853741	0.000220401
Chr1g0364661	Zbsp002083	0.978546673	0.000685431
Chr1g0364661	Waptp02347	0.988047783	0.00021343
Chr1g0364661	Zblp002396	0.989517097	0.000164261
Chr1g0364661	Zmjp001877	0.982247676	0.00046992
Chr1g0364661	Zasp002650	0.982932135	0.000434482
Chr1g0364661	Zbzp001964	0.988678204	0.000191549
Chr1g0364661	Lmqp001551	0.988385681	0.000201555
Chr1g0364661	Zmmp002642	0.989391739	0.000168206
Chr1g0364661	Zbpp001841	0.990700915	0.000129307
Chr1g0364661	Lmjp001367	0.987109937	0.00024816
Chr1g0364661	mws0044	0.964783708	0.001838443
Chr1g0364661	MWSHY0051	-0.989687435	0.000158975
Chr3g0468161	Zbjp001957	0.979919201	0.000600809
Chr3g0468161	Zbsp002083	0.987810561	0.000221968
Chr3g0468161	Waptp02347	0.984490874	0.000358934
Chr3g0468161	Zblp002396	0.987226976	0.000243683
Chr3g0468161	Zmjp001877	0.9907111	0.000129025
Chr3g0468161	Zasp002650	0.99070523	0.000129188
Chr3g0468161	Zbzp001964	0.988362501	0.000202359
Chr3g0468161	Lmqp001551	0.986615847	0.000267505
Chr3g0468161	Zmmp002642	0.98503881	0.000334081
Chr3g0468161	Zbpp001841	0.987258385	0.000242489
Chr3g0468161	Lmjp001367	0.988942486	0.000182727
Chr3g0468161	MWSHY0029	0.960471037	0.002312926
Chr3g0468161	mws0044	0.989627442	0.000160827
Chr3g0468161	MWSHY0051	-0.973329152	0.001057515
Chr6g0308731	Zbjp001957	-0.990014185	0.000149077
Chr6g0308731	Zbsp002083	-0.985678335	0.000306196

Continued

Chr6g0308731	Waptp02347	-0.992935913	7.46757E-05
Chr6g0308731	Zblp002396	-0.989967348	0.000150476
Chr6g0308731	Zmjp001877	-0.986960465	0.000253936
Chr6g0308731	Zasp002650	-0.987237116	0.000243297
Chr6g0308731	Zbzp001964	-0.988867306	0.000185215
Chr6g0308731	Lmqp001551	-0.990753221	0.000127859
Chr6g0308731	Zmmp002642	-0.990812347	0.000126232
Chr6g0308731	Zbpp001841	-0.989888416	0.000152849
Chr6g0308731	Lmjp001367	-0.988811254	0.000187082
Chr6g0308731	MWSHY0029	-0.929510163	0.0072781
Chr6g0308731	mws0044	-0.958962239	0.002491591
Chr6g0308731	MWSHY0051	0.982450226	0.000459289
Chr1g0360311	Zbjp001957	0.996516493	1.81811E-05
Chr1g0360311	Zbsp002083	0.994022358	5.34915E-05
Chr1g0360311	Waptp02347	0.99612068	2.25445E-05
Chr1g0360311	Zblp002396	0.998492692	3.40625E-06
Chr1g0360311	Zmjp001877	0.993173021	6.97524E-05
Chr1g0360311	Zasp002650	0.99344591	6.42934E-05
Chr1g0360311	Zbzp001964	0.995213776	3.43071E-05
Chr1g0360311	Lmqp001551	0.99797993	6.1169E-06
Chr1g0360311	Zmmp002642	0.998186557	4.92988E-06
Chr1g0360311	Zbpp001841	0.997292586	1.09852E-05
Chr1g0360311	Lmjp001367	0.998706324	2.50931E-06
Chr1g0360311	MWSHY0029	0.953839916	0.003146952
Chr1g0360311	mws0044	0.994657991	4.27294E-05
Chr1g0360311	MWSHY0051	-0.99358625	6.15724E-05
Chr4g0440111	Zbjp001957	0.998271892	4.47696E-06
Chr4g0440111	Zbsp002083	0.988831469	0.000186408
Chr4g0440111	Waptp02347	0.993327433	6.66362E-05
Chr4g0440111	Zblp002396	0.996142699	2.22895E-05
Chr4g0440111	Zmjp001877	0.984608758	0.000353512
Chr4g0440111	Zasp002650	0.985004037	0.000335632
Chr4g0440111	Zbzp001964	0.989005096	0.000180667
Chr4g0440111	Lmqp001551	0.995633667	2.85557E-05
Chr4g0440111	Zmmp002642	0.996993701	1.35432E-05
Chr4g0440111	Zbpp001841	0.995783795	2.66271E-05
Chr4g0440111	Lmjp001367	0.995126273	3.55719E-05
Chr4g0440111	MWSHY0029	0.935175576	0.006167106
Chr4g0440111	mws0044	0.990822875	0.000125943
Chr4g0440111	MWSHY0051	-0.997816482	7.14642E-06
Chr5g0012431	Zbjp001957	-0.997647214	8.29689E-06
Chr5g0012431	Zbsp002083	-0.98869136	0.000191105
Chr5g0012431	Waptp02347	-0.994534593	4.47244E-05

Continued

Chr5g0012431	Zblp002396	-0.997172379	1.19819E-05
Chr5g0012431	Zmjp001877	-0.986686746	0.000264684
Chr5g0012431	Zasp002650	-0.987194796	0.00024491
Chr5g0012431	Zbzp001964	-0.991979873	9.62257E-05
Chr5g0012431	Lmqp001551	-0.996410753	1.93009E-05
Chr5g0012431	Zmmp002642	-0.997724545	7.76065E-06
Chr5g0012431	Zbpp001841	-0.995429902	3.1281E-05
Chr5g0012431	Lmjp001367	-0.996300825	2.05005E-05
Chr5g0012431	MWSHY0029	-0.937852457	0.005673459
Chr5g0012431	mws0044	-0.989320763	0.00017046
Chr5g0012431	MWSHY0051	0.998261418	4.53137E-06

Note: MWSHY0029, quercetin; mws0044, taxifolin; MWSHY0051, kaempferol; Zbjp001957, cyanidin 3,5-O-diglucoside; Zbsp002083, pelargonin 3,5-O- diglucoside; Waptp02347, peonidin 3-O-sophoroside; Zmjp001877, cyanidin 3-O-beta-D- sambubioside; Zblp002396, peonidin 3-O-glucoside; Lmqp001551, cyanidin 3,3',5-tri-O-glucoside; Zbzp001964, cyanidin-3-di-glucoside-5-glucoside; Zasp002650, cyanidin-3-O-galloyl-galactoside; Zmmp002642, cyanidin-3-O-(6''-O-feruloyl) glucoside; Lmjp001367, cyanidin 3-O-(beta-D-xylosyl-(1->2)-beta-D-galactoside); Zbpp001841, peonidin-3,5-O-diglucoside.
